# Leveraging Natural Killer Cell Innate Immunity against Hematologic Malignancies: From Stem Cell Transplant to Adoptive Transfer and Beyond

**DOI:** 10.3390/ijms24010204

**Published:** 2022-12-22

**Authors:** Chenyu Lin, Mitchell E. Horwitz, Lindsay A. M. Rein

**Affiliations:** Division of Hematologic Malignancies & Cellular Therapy, Duke University, Durham, NC 27710, USA

**Keywords:** natural killer cells, stem cell transplantation, adoptive transfer, chimeric antigen receptor, bispecific antibodies, cytokines, hematologic malignancies

## Abstract

Numerous recent advancements in T-cell based immunotherapies have revolutionized the treatment of hematologic malignancies. In the race towards the first approved allogeneic cellular therapy product, there is growing interest in utilizing natural killer (NK) cells as a platform for off-the-shelf cellular therapies due to their scalable manufacturing potential, potent anti-tumor efficacy, and superior safety profile. Allogeneic NK cell therapies are now being actively explored in the setting of hematopoietic stem cell transplantation and adoptive transfer. Increasingly sophisticated gene editing techniques have permitted the engineering of chimeric antigen receptors, ectopic cytokine expression, and tumor recognition signals to improve the overall cytotoxicity of NK cell therapies. Furthermore, the enhancement of antibody-dependent cellular cytotoxicity has been achieved through the use of NK cell engagers and combination regimens with monoclonal antibodies that act synergistically with CD16-expressing NK cells. Finally, a greater understanding of NK cell biology and the mechanisms of resistance have allowed the preclinical development of NK checkpoint blockade and methods to modulate the tumor microenvironment, which have been evaluated in early phase trials. This review will discuss the recent clinical advancements in NK cell therapies in hematologic malignancies as well as promising avenues of future research.

## 1. Introduction

Natural killer (NK) cells are an integral component of the innate immune system, known to play important roles in the immune surveillance of virally-infected and transformed cells [1]. Over the past decade, significant strides have been made in harnessing the cytotoxic functions of these vital innate effector cells against various hematologic malignancies. Knowledge acquired from early studies of NK cells in the setting of allogeneic hematopoietic stem cell transplantation (HSCT) laid the groundwork for the subsequent development of adoptive transfer strategies [2,3]. The recent success of autologous chimeric antigen receptor T cell (CAR-T) immunotherapy, along with their limitations, have further reinvigorated interest in ex vivo gene-edited immune effector cellular therapies utilizing novel allogeneic immune cell platforms such as NK cells [4,5]. Many of these innovative therapeutic approaches have now been translated from the bench into early phase clinical trials [6,7]. The purpose of this article is to review advancements in NK-based cellular therapy approaches for hematologic malignancies, with a focus on strategies that are being actively investigated in the clinical setting.

### 1.1. NK Cell Biology

NK cells are characterized as CD3^−^CD56^+^ large granular lymphocytes consisting of two broad subtypes: the naïve CD56^bright^CD16^dim^ subsets and the more common and mature CD56^dim^CD16^+^ subsets [8]. These cells are broadly distributed across the peripheral blood as well as the bone marrow, lymph nodes, spleen, and liver [9,10]. They partake in several critical roles in the human innate immune system, including surveillance and cytotoxic functions against tumor cells [1]. Long-term follow-up demonstrated that low NK cell cytotoxic activity was associated with increased cancer risk over time [11]. In addition, the activation and maturation status of NK cells have shown prognostic significance in leukemias including B- and T-cell acute lymphoblastic leukemia (ALL) [12].

NK cells can recognize and lyse tumor targets in a major histocompatibility complex (MHC)-unrestricted manner without the need for prior antigen sensitization. Effector function is modulated through a complex array of activating and inhibitory signals present on the effector cell surface, allowing differentiation of the body’s own healthy cells from transformed or foreign cells [13]. Inhibitory receptors include certain C-type lectin-like receptors (e.g., NK group 2A, or NKG2A, which recognize human leukocyte antigen E), inhibitory killer Ig-like receptors (KIRs), and leukocyte Ig-like receptors (LILRs) [14,15,16]. One prominent inhibitory interaction involves the recognition of self-MHC molecules on the target cells. Cancer cells that downregulate MHC class I expression to elude CD8^+^ T cell cytotoxicity will instead become susceptible to NK cell targeting, which distinguish these MHC-deficient cells as foreign [14,17]. In addition, non-MHC-specific receptors such as programmed cell death protein-1 (PD-1) and cytotoxic T-lymphocyte associated protein-4 (CTLA-4) can also have inhibitory properties on NK cell function [18]. Activating receptors include activating C-type lectin-like receptors (e.g., NK group 2D, or NKG2D, which binds inducible stress ligands), natural cytotoxicity receptors (e.g., NK p46-related protein, or NKp46), activating KIRs, and Fc-γ receptor IIIA (i.e., CD16) [19]. Importantly, CD16 binds to the Fc portion of immunoglobulin G (IgG) to activate antibody-dependent cellular cytotoxicity (ADCC) [20].

### 1.2. Mechanisms of NK Cytotoxicity

**Figure 1:**
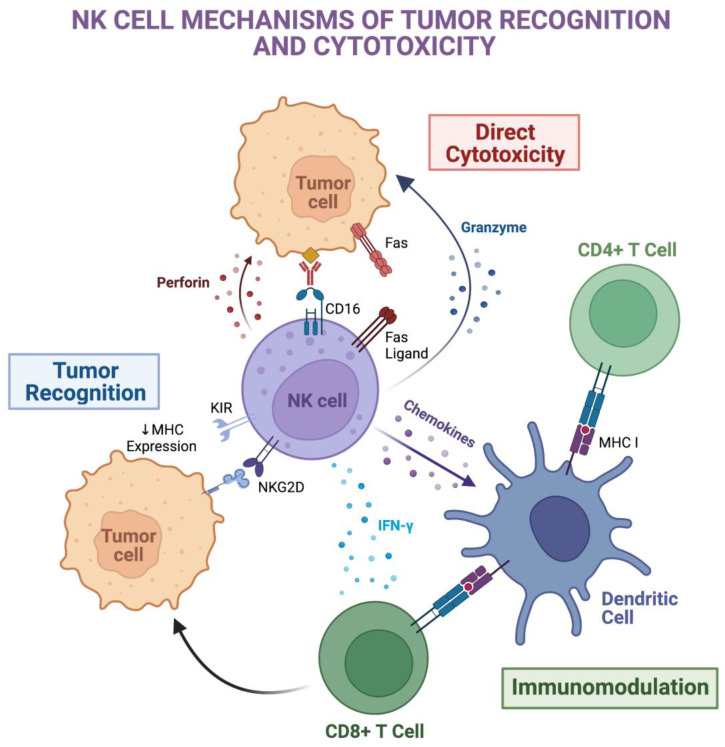
Mechanisms of anti-tumor recognition and cytotoxicity by NK cells. NK cell recognition of tumor cells is mediated by a complex array of activating and inhibitory receptors present on the cell surface. The C-type lectin-like receptor, NKG2D, is able to recognize stress-induced tumor ligands. In addition, tumor cells frequently downregulate MHC expression to avoid detection by CD8+ T cells, which makes them prone to NK cell killing due to the lack of KIR inhibitory signaling. Upon recognition of the tumor cell, mechanisms of direct cytotoxicity include binding of programmed cell death receptors (e.g., Fas ligand-Fas), antibody-dependent cellular cytotoxicity mediated by CD16 binding to the Fc portion of tumor-bound antibodies, and release of perforin and granzyme granules. NK cells may also exert immunomodulatory effects on nearby immune cells such as antigen presenting cells and effector T cells to enhance the overall anti-tumor response. Created with BioRender.

NK cells are able to exert their cytotoxic function through a number of cell-killing mechanisms (Figure 1):

(1) The release of lytic granules such as perforin and granzyme leading to cell lysis [21].
(2) The engagement of Fas ligand or tumor necrosis factor related apoptosis-inducing ligand (TRAIL) targets cell death receptors leading to programmed cell death [21].
(3) Antibody-dependent cellular cytotoxicity mediated by CD16 binding to an IgG Fc segment on the opsonized tumor cell [22].

Primary CD56^bright^ NK cells principally utilize Fas ligand binding, while CD56^dim^ and certain immortalized NK cell lines rely on perforin and granzyme-mediated cytotoxicity [23,24]. NK cells also possess indirect anti-tumor functions through cross-talk with other bystander immune cells. NK release of cytokines, such as interferon-γ (IFN-γ) and tumor necrosis factor α (TNF-α), and chemokines, including chemokine ligand 3 (CCL3) and C-X-C motif chemokine ligand 8 (CXCL8), can modulate and recruit nearby antigen presenting cells [16]. Furthermore, they are capable of transforming CD8^+^ T cells into cytotoxic T lymphocytes and activating macrophage killing to augment the overall anti-tumor response [25].

## 2. NK Cells in Hematopoietic Stem Cell Transplantation

Some of the earliest clinical evidence of the robust anti-tumor properties of NK cells against hematologic malignancies were observed in the context of HSCT [2]. Among the lymphoid subsets, NK cells typically feature the most rapid reconstitution after HSCT and contribute significantly to the graft-versus-tumor effect and viral immune responses in the early post-transplant period [26]. Faster NK cell recovery has also been associated with improved outcomes after autologous HSCT for both non-Hodgkin lymphoma (NHL) and multiple myeloma (MM) [27,28]. Recent efforts have attempted to enhance the graft-versus-tumor effect of allogeneic HSCT through infusions of human leukocyte antigen (HLA)-mismatched NK cells.

### 2.1. KIR Mismatch in Allogeneic HSCT

While inhibitory KIRs on NK cells are known to recognize HLA class I molecules, donor NK cells from an allogeneic HSCT do not bind to recipient HLA I and can circumvent the suppressive inhibitory signals. Therefore, multiple studies have evaluated KIR mismatch in the graft-versus-host direction and were able to demonstrate a reduction in the risk of cancer relapse after allogeneic HSCT [29,30]. In a seminal study by Ruggeri et al., 57 patients with acute myeloid leukemia (AML) undergoing T cell depleted haploidentical HSCT carrying these alloreactive NK cells had a significantly lower five-year risk of disease relapse (0%) compared to those without KIR ligand incompatibility (75%), suggesting a considerable impact of KIR mismatch on the graft-versus-leukemia effect [2]. Similarly, in a larger study of 130 patients undergoing unrelated donor HSCT for hematologic malignancies, KIR mismatch resulted in significantly improved 4.5-year overall survival (87% vs. 48%) and disease-free survival (87% vs. 39%), as well as numerically reduced relapse rates (6% vs. 21%) [3].

While the impacts of specific HLA-KIR genotypes are still being explored, one study has demonstrated that patients carrying an HLA-C1 allele who receive cord blood grafts with HLA-C1-KIR2DL2/L3/S2 genotypes have lower relapse rates compared to those who receive grafts lacking HLA-C1 or KIR2DS2 [31]. Similarly, patients with HLA-C2/C2 alleles have lower relapse rates using grafts carrying the HLA-C2-KIR2DL1/S1 genotype [31]. In a separate study of allogeneic HSCT for chronic myelogenous leukemia (CML), patients with the HLA-Bw4 allele who received grafts with KIR3DS1 also had reduced rates of molecular and hematologic relapse [32]. Among children with leukemia undergoing haploidentical transplants, donor-derived KIR2DL2/3+ NK cells showed reduced alloreactivity against HLA-C2 leukemia cells, while KIR2DL1+ NK cells had robust killing against HLA-C1 leukemia cells [33]. Together, these results demonstrated that consideration of the specific HLA-KIR genotype is important in optimizing KIR mismatch alloreactivity.

The potential beneficial effects of KIR mismatch are not limited to reduction of disease relapse. Unlike αβ T cells, animal and clinical studies have demonstrated that large infusions of allogeneic NK cells do not precipitate graft-versus-host disease (GVHD) likely due to their MHC-unrestricted recognition mechanisms [2,34]. In fact, KIR-HLA mismatch in the graft-versus-host direction may protect allogeneic HSCT recipients against the development of GVHD, possibly through elimination of host antigen-presenting dendritic cells and alloreactive GVHD-mediating T cells [2,34,35].

### 2.2. HLA-Mismatched NK Cell Infusions with Allogeneic HSCT

The clinical translation of these observations for allogeneic HSCT has primarily taken the form of peri- and post-transplant infusions of mismatched NK cells in an effort to improve transplant outcomes. One phase II trial evaluated NK cell infusions from haploidentical donors after lymphodepletion using a cyclophosphamide-based regimen in patients with AML or myelodysplastic syndrome (MDS) relapsed after allogeneic HSCT [36]. Eight patients received mismatched NK cells at a median dose of 10.6 × 10^6^ cells/kg, with two patients achieving a transient complete response (CR). However, in vivo persistence was a major limitation and no haploidentical NK cells were detected even at 3 days post-infusion, possibly related to insufficient cell doses, inadequate cytokine support, or suppression from T regulatory (Treg) and myeloid-derived suppressor cell populations.

A subsequent phase I/II study investigated peri-transplant infusions of membrane-bound IL-21 expanded NK cells in patients with myeloid malignancies undergoing haploidentical HSCT after conditioning with fludarabine, melphalan, and 2 Gy of total body irradiation as well as prophylaxis with post-transplant cyclophosphamide (PTCy) [37]. These NK cells were derived from the original HSCT donors who were selected with a preference for NK alloreactivity in the graft-versus-host direction. This expansion method was able to achieve a ~3000-fold expansion, reaching the target cell dose of 1 × 10^8^ cells/kg/dose in 12 of 13 patients. The 2-year relapse rate was 4% in the study cohort compared to 38% from a historical control, with observations of increased 30-day NK cell frequency correlating with NK cell dose. As PTCy is increasingly utilized in the prevention of GVHD after haploidentical and unrelated donor HSCT, their impact on NK cell proliferation must also be considered. The ablation of alloreactive NK cells by PTCy may lead to loss of the benefit of KIR mismatch on relapse rates [38]. The timing of peri-transplant NK cell infusions will need to take into account PTCy elimination of rapidly-proliferating lymphoid cells in the early transplant period.

Ongoing attempts to capitalize on KIR mismatch in HSCT have led to findings that specific combinations of KIR and HLA genotypes can benefit NK cell function and improve relapse and survival after cord blood transplantation for myeloid and lymphoid malignancies [31]. This rationale is being investigated in an ongoing phase II trial of personalized cord blood transplants selected based on HLA-KIR typing followed by adoptive transfer of donor NK cells at 1–3 months post-transplant (NCT02727803). Additional efforts have involved the use of NK cells in donor lymphocyte infusions (DLI). In one study of pediatric and young adults with post-HSCT relapse of AML, standard DLI in combination with memory-like NK DLI led to remissions in four of eight evaluable patients with evidence of NK cell persistence at 3 months [39]. Other studies leveraging donor-derived NK cells with allogeneic HSCT are currently ongoing (NCT02452697, NCT03300492, NCT03068819).

### 2.3. NK Cells for Post-Transplant Infections

NK cells provide innate immunity and defense against a number of viral infections. Infusions of memory-like NK cells have been associated with a lower incidence of post-transplant cytomegalovirus reactivation [40]. Similarly, NK cells exhibit cytotoxic activity against the SARS-CoV-2 virus [41,42]. A recent preclinical study evaluated the adoptive transfer of allogeneic NK cells with soluble interleukin (IL)-15 and engineered chimeric antigen receptors specific to SARS-CoV-2 viral antigens, demonstrating a proof of concept for this off-the-shelf approach for severe coronavirus disease-19 (COVID-19) infections [43]. These findings may have implications for the management of post-transplant infections but have not yet been investigated in the clinical setting. A comprehensive coverage of this topic is beyond the scope of this article but has been reviewed in other sources [44,45,46].

### 2.4. NK Cell Therapies in Autologous HSCT

NK cell infusions have also been explored in the context of autologous HSCT. A first-in-human phase I clinical trial evaluated cord blood-derived NK cells infused on day-5 in patients with multiple myeloma receiving melphalan conditioning and autologous HSCT [47]. Ten of 12 patients achieved a very good partial response (VGPR), with no reports of GVHD. Donor NK cells showed strong expression of NKG2D and were detected at up to 26 days in vivo. In a similar study, haploidentical NK cells were infused in the first month post-auto HSCT as a consolidative step for multiple myeloma [48]. This approach also demonstrated a favorable safety profile, though there was no effect on relapse or survival when compared to a historical cohort. Finally, a phase I study assessed an off-the-shelf NK cell product derived from human placental CD34+ cells with and without recombinant IL-2 on day 14 post-autologous HSCT [49]. This resulted in at least VGPR in 10 of 12 multiple myeloma patients without a significant impact on immune reconstitution kinetics. Additional ongoing trials evaluating allogeneic NK cells following autologous HSCT for NHL and multiple myeloma include NCT03019640 and NCT04309084. Ongoing clinical trials of NK cell therapies with and without HSCT are summarized in Table 1.

## 3. Adoptive Transfer of NK Cells

Initial attempts at adoptive transfer of autologous NK cells have been largely disappointing. In vivo expansion using high dose IL-2 after autologous HSCT resulted in significant adverse events, with many patients experiencing severe capillary leak syndrome [50,51]. Ex vivo expanded autologous NK cells as well as in vivo expansion via low dose IL-2 were able to achieve expansion with relatively fewer toxicities, but have demonstrated limited anti-tumor potency [52,53]. The lack of efficacy with autologous NK cell therapies has been hypothesized to be due to the recognition of self-HLA antigens by inhibitory receptors, dysfunctional and exhausted NK cell phenotypes pre-collection, and IL-2 upregulation of Treg inhibitory properties [54,55,56]. These findings have prompted a greater interest in adoptive transfer from allogeneic sources, particularly with the unique advantage of NK cell platforms in not causing GVHD. Furthermore, experiences in HSCT have confirmed the anti-tumor effects of mismatched NK cells, and thus efforts in the field have focused on expanding allogeneic transfer approaches to outside of the transplant setting.

Allogeneic NK cells used in adoptive transfer may be derived from a number of sources, including peripheral blood, umbilical cord blood, immortalized cell lines, and induced pluripotent stem cells. Methods to enhance cytotoxicity, promote expansion, and gene editing of chimeric antigen receptors are being actively investigated to improve the efficacy of NK cell therapies.

### 3.1. Peripheral Blood and Umbilical Cord Blood

The majority of adoptive transfer approaches using allogeneic sources have relied on procuring NK cells from the peripheral blood and umbilical cord blood. NK cells amount to only 10% of circulating lymphocytes in the peripheral blood and approximately 30% in cord blood, thus necessitating ex vivo expansion techniques to permit adequate cell collection [57,58]. While peripheral blood requires collection from individual donors, cord blood can be obtained off-the-shelf from dedicated cord blood banks, making it a suitable platform for allogeneic approaches. However, compared to peripheral blood, the relatively immature NK cells taken from cord blood may possess reduced cytotoxic potential as they tend to express lower levels of perforin and granzyme as well as more inhibitory NKG2A, though the latter may be overcome by cytokine stimulation [59,60].

In an important early study by Miller et al., 19 AML patients received haploidentical NK cell infusions at doses up to 2 × 10^7^ cells/kg with or without modified IL-2 injections, with five patients achieving a morphologic CR [61]. Three lymphodepletion regimens were evaluated in this trial, including two low dose outpatient regimens and a high dose inpatient regimen, the latter of which consisted of intravenous fludarabine 25 mg/m^2^ and cyclophosphamide 60 mg/kg for five days. Notably, this study was among the first to demonstrate in vivo expansion of adoptively transferred NK cells, which was observed only with the higher dose preparative course. These findings reinforce the importance of establishing an appropriate immunologic niche with adequate depletion of T lymphocytes to allow for optimal expansion of infused NK cells. Furthermore, a rise in IL-15 levels correlated with the intensive preparative regimen, suggesting a potential candidate for cytokine-supported expansion as an alternative to IL-2.

Subsequent reports on adoptive transfers of NK cells have further confirmed the safety and feasibility of this approach, with conditioning-related myelosuppression being the most common significant adverse event [62,63,64,65,66,67]. Reassuringly, GVHD has not been observed in the absence of an allogeneic HSCT, though persistence and efficacy have been variable across the studies. Early phase trials have also evaluated novel ex vivo priming strategies utilizing leukemia cell lysate to activate NK cells, permitting in vivo expansion even in the absence of cytokine support [67,68]. Finally, while NK cells have traditionally been considered a part of the innate immune system, recent preclinical studies have suggested that certain NK subsets may differentiate into a memory-like phenotype with enhanced effector function after pre-activation using a cytokine cocktail [69,70]. A phase I study of haploidentical memory-like NK cell infusions in relapsed and refractory AML yielded an overall response rate (ORR) of 55% among evaluable patients [71]. These memory-like NK cells also demonstrated significant in vivo proliferation and persistence, with improved cytotoxicity and IFN-γ secretion. More recent studies of memory-like NK cells in the HSCT setting have confirmed their expansion and persistence capabilities, suggesting a promising approach for future adoptive transfer endeavors [39,72].

The clinical experiences with adoptive transfer of NK cells derived from peripheral blood and cord blood have confirmed an excellent safety profile but offered conflicting reports on efficacy. However, as these cells ultimately come from individual donors, there remains unpredictable inter-donor heterogeneity between batches of expanded cells leading to inconsistent effector function.

### 3.2. Immortalized Cell Lines and Induced Pluripotent Stem Cells (iPSCs)

The known limitations of peripheral blood and cord blood-derived NK cells have motivated efforts to develop more reliable platforms for NK cell therapies. NK-92 is an immortalized CD56^bright^CD16^−^ NK cell line collected from a patient with a rare NK cell lymphoma. The benefits of using NK-92-based cellular therapies are manyfold. It possesses excellent cytotoxic function related to its overexpression of several activating receptors, down-regulation of inhibitory receptors, and high expression of lytic granules [73]. Moreover, the cell line is highly proliferative, easily acquired, and importantly, homogeneous in phenotype, allowing for a consistent and reproducible platform for translation of advanced cellular therapies [74].

Multiple phase I clinical trials have been performed investigating irradiated NK-92 cell infusions in patients with various hematologic malignancies [75,76]. The studies reported few treatment-related toxicities but only modest efficacy, possibly related to limited persistence as one study showed no detectable NK-92 cells beyond 15 min post-infusion [75]. Due to their derivation from an NK cell neoplasm, NK-92 cells must be irradiated to prevent malignant proliferation, thus limiting their capacity for in vivo expansion and persistence [77]. Other limitations of NK-92 cells include a susceptibility to targeting by host NK cells, high dependence on exogenous IL-2 for survival, and the absence of CD16 [73,78]. To this end, high-affinity NK cells (haNKs) have been developed by engineering NK-92 cells to endogenously express IL-2 along with high-affinity CD16 receptors carrying the 158V polymorphism, allowing for potent ADCC function [79]. This method is being actively explored in various solid tumors, though the QUILT 3.061 study for lymphoma (NCT04052061) is currently on hold. There also exists preclinical evidence that haNKs may have synergy with monoclonal antibody therapies such as rituximab in follicular lymphoma and daratumumab in multiple myeloma, but these approaches still need to be explored in the clinical setting [80,81].

Induced pluripotent stem cells (iPSCs) are derived from mature somatic cells that have been reprogrammed into a state of pluripotent stemness through ectopic expression of transcription factors involved in the maintenance of pluripotency [82]. Similar to NK-92, iPSC-derived NK cells (iPSC-NK) are highly proliferative and clonal, but they lack a malignant etiology and thus do not require irradiation. Furthermore, iPSC clones can be selected and modified for anti-tumor potential, with subsequent expansion allowing for a homogeneous product [83]. While CD16 expression is also low on iPSC-NK cells, this deficit can be corrected with genetic modification [84]. Interim results from a phase I dose-escalation study have been reported for FT516, an allogeneic iPSC-derived NK cell product expressing a high-affinity CD16 and endogenous IL-2, though the results have not yet been formally published [85]. Four of nine evaluable patients with AML reportedly experienced a response, with one patient proceeding to an allogeneic HSCT. Preliminary results of FT516 in combination with rituximab for relapsed or refractory B cell lymphomas have also been reported, showing a 75% ORR after enrolling the first six patients [86].

### 3.3. NK Chimeric Antigen Receptors (CAR-NK)

Chimeric antigen receptors (CAR) are engineered receptors that allow for redirection of effector cell specificity to a predetermined target antigen. The structure of the commercially available second-generation CARs are comprised of three major components: an extracellular single-chain variable fragment specific to a tumor antigen; a transmembrane domain; and an intracellular signaling complex including a costimulatory domain (e.g., 4-1BB, CD28) [87]. There are currently six autologous CAR-T cell products approved by the U.S. Food and Drug Administration for the treatment of hematologic malignancies. However, despite the success of commercial CAR-T therapies, there remains several important limitations. Prolonged manufacturing times have restricted the use of autologous CAR-T in patients with rapidly progressive disease [88]. There are also high rates of CAR-associated toxicities, including cytokine release syndrome (CRS) and immune effector cell-associated neurotoxicity syndrome (ICANS) [4,5]. Finally, CAR-T efficacy may be impacted by exhausted phenotypes, low quantities of the starting product, or resistance mechanisms such as tumor antigen escape [87]. Many of these limitations may be overcome by adopting an allogeneic strategy, but the αβ T cell platform used in conventional CAR-T requires additional gene editing to prevent GVHD [89]. The MHC-unrestricted antigen recognition mechanism of NK cells offers a distinct advantage over αβ T cells as an allogeneic platform by avoiding GVHD [34,90]. In addition, native NK cell receptors can recognize tumor antigens independent of CAR targeting, ameliorating antigen escape mechanisms [90].

Early phase CAR-NK studies have demonstrated minimal rates of CRS, ICANS, or GVHD, establishing the safety and feasibility of this approach [7,91]. A phase I study of allogeneic iPSC-derived CD19 CAR-NK with high affinity CD16 in combination with an anti-CD20 monoclonal antibody enrolled 20 patients with relapsed or refractory NHL, reporting only two cases of low-grade CRS and no ICANS or GVHD [7]. The significantly lower incidence of CAR-associated toxicities compared to commercial CAR-T is likely due to the differing cytokine profiles between NK cells and T cells, making CAR-NK products less prone to CRS and ICANS [90,92]. Moreover, the high ORR of 73% observed in this study may suggest potential synergy between the engineered high affinity CD16 and the exogenous anti-CD20 antibody enhancing the natural ADCC response, allowing for alternative avenues of cytotoxicity outside of CAR signaling. Another first-in-human phase I trial infused an allogeneic NK-92-derived CD33 CAR-NK product in 3 patients with relapsed AML following salvage chemotherapy, observing only grade I CRS [91]. The authors specifically noted the much lower costs of production when compared to CAR-T, highlighting the potential of these iPSC and NK-92 cells to support scalable manufacturing platforms.

A recent high-profile study by Liu et al. investigated HLA-mismatched, cord blood-derived CD19 CAR-NK cells expressing IL-15 and an inducible cas-9 safety switch in patients with relapsed or refractory CD19^+^ NHL [6]. This phase I/II clinical trial treated 11 patients at total doses up to 1 × 10^7^ cells/kg, yielding an ORR of 73% and, impressively, a CR rate of 64%. All responses were rapid and occurred within the first month. A small contamination (0.01%) of CAR-T was detected but not expected to have impacted the response significantly. Similar to prior reports, there were no cases of CRS, ICANS, or GVHD. Remarkably, quantitative real-time polymerase chain reaction (PCR) of the CAR-NK vector transgene demonstrated expansion starting at 3 days and low-level persistence for at least 12 months after infusion, which may be related to the transduced IL-15 expression [93]. The excellent safety profile of this HLA-mismatched approach and the ability to generate over 100 doses of CD19 CAR-NK cells from a single unit of cord blood again reinforces the potential to develop this platform into a cost-effective off-the-shelf therapy [93].

Future directions in CAR-NK research will involve novel targets such as fms-like tyrosine kinase 3 (FLT3) for leukemia as well as CD138 and CCND subset 1 (CS1) for multiple myeloma [94,95,96]. Combinations of CAR-NK and CAR-T therapies have been assessed in vitro, though CAR-NK cells may impair proliferation of CAR-T [97]. Novel CAR constructs including the use of NKG2D receptors, fourth generation CARs engineered with immunomodulatory cytokines to recruit bystander immune cells, and dual CAR-NK targeting are all being actively developed [98,99,100]. More NK-specific co-stimulatory domains are also needed, as current structures borrow from T cell signaling complexes. In particular, DNAX-activating protein 10 (DAP10), DNAX-activating protein 12 (DAP12), and NKG2D-2B4ζ have been identified as NK-specific signaling domains, which may be able to further optimize CAR-NK function [101,102,103]. Finally, engineered T cell receptors (TCR) are being developed for NK cell therapies [104]. While CARs utilizing a single chain variable fragment are limited to recognition of surface proteins, TCRs are able to respond to a variety of MHC-bound intracellular peptides [105]. Preclinical studies have demonstrated the feasibility of genetically engineering functional TCRs into NK cell platforms such as NK-92 [104].

### 3.4. Role of Cell Dose in NK Cell Therapy

The role of cell dose has been explored in the adoptive transfer of NK cells. While there is considerable variability in the amount of alloreactive NK cells that are procured and subsequently transferred, one study showed that cell products containing at least 8% alloreactive NK cells (and a threshold of at least 2 × 10^5^/kg alloreactive donor NK cells) may result in lower relapse rates in AML [106,107]. In the long-term follow-up of this study, anti-tumor efficacy maintained its correlation with the functional cell dose of alloreactive NK cells [108]. Similarly, clinical trials of CAR-NK products in lymphoma have suggested that higher cell doses may improve elimination of minimal residual disease, though a minimum threshold was not determined [6]. Further investigations of optimal NK cell dosing are underway [109]. Notably, allogeneic NK cell therapies retain their favorable safety profile despite increases in cell dose, with the majority of early phase trials not reaching the maximum tolerated dose [110,111,112]. The limited toxicities observed in these dose escalation trials did not appear to be dose dependent, suggesting the potential for the infusion of higher doses of NK cells to optimize efficacy.

## 4. Moving beyond CARs: Strategies to Enhance NK Cell Therapy Efficacy

**Figure 2:**
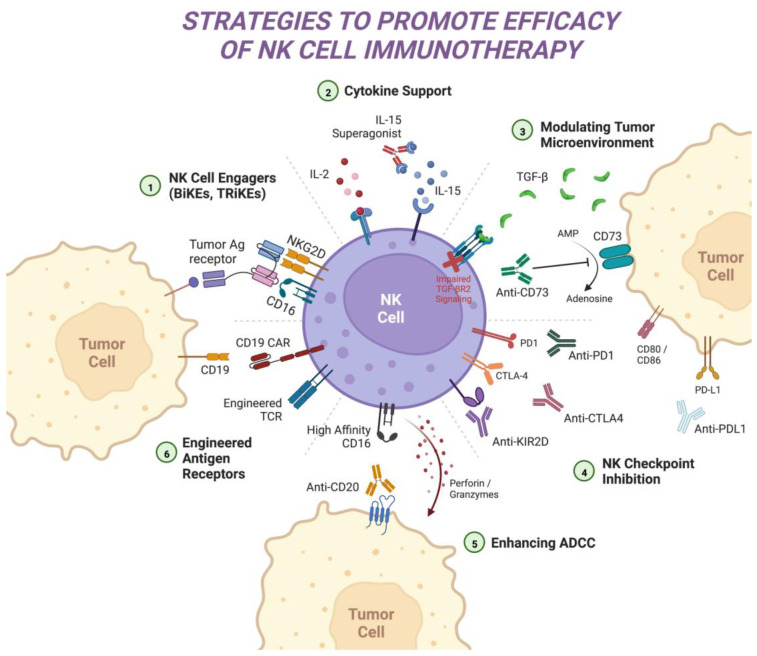
Strategies to promote anti-tumor effector function for NK cell immunotherapy. (1) NK cell engagers include bispecific (BiKEs) and trispecific antibodies (TRiKEs) that bring tumor antigens and supportive cytokines into proximity with NK cell receptors. (2) Supportive cytokine-mediated approaches include the use of IL-2 and IL-15 to promote NK cell expansion and cytotoxicity. (3) Impairing TGF-β signaling and preventing buildup of adenosine in the tumor microenvironment can reduce local suppressive effects on NK cells. (4) Blockade of immune checkpoints (PD-1, PD-L1, CTLA4) and inhibitory NK receptors (KIR2D) may circumvent NK exhaustion and improve anti-tumor function. (5) The addition of monoclonal antibodies and engineering of high affinity CD16 receptors promote the CD16-Fc ligand binding interaction, leading to enhanced ADCC. (6) Engineered selective antigen receptors such as CARs and TCRs can redirect NK cells towards specified tumor antigens and increase cytotoxicity. NK: natural killer, BiKE: bispecific killer engagers, TRiKE: trispecific killer engagers, Ag: antigen, NKG2D: natural killer group 2 member D, IL: interleukin, TGF-β: transforming growth factor beta, AMP: adenosine monophosphate, PD-1: programmed cell death protein 1, CTLA-4: cytotoxic T-lymphocyte associated protein 4, KIR2D: killer immunoglobulin-like receptor group 2 member D; PD-L1: programmed death-ligand 1, ADCC: antibody-dependent cellular cytotoxicity, TCR: T cell receptor, CAR: chimeric antigen receptor. Created with BioRender.

While the advent of CAR engineering has improved the efficacy of NK cell adoptive transfer, CAR-NK has not yet achieved the success seen with CAR-T therapy in the hematologic malignancies. Therefore, alternative novel approaches are being actively investigated (Figure 2). Limited NK cell persistence continues to present a barrier to durable responses, and further fine-tuning of cytokine expression strategies will be necessary. Mechanisms of NK cell resistance including effector cell exhaustion and suppression from the tumor microenvironment (TME) also need to be addressed. Additional studies are evaluating synergistic combinations with monoclonal antibodies that can enhance ADCC functions as well as with other antineoplastic agents.

### 4.1. Cytokine-Mediated Approaches

Cytokine support is a commonly applied strategy to enhance anti-cancer function and promote NK cell persistence. Various regulatory cytokines have been shown to modulate the proliferation and activation of NK cells, including IL-2, IL-12, IL-15, and IL-21 [113,114,115,116]. IL-2 is known to be integral to the survival of NK cells, though toxicities may be significant at higher doses and efficacy is limited by upregulated Treg cells [50,56]. In a phase II trial, 57 patients with refractory AML were given lymphodepletion with fludarabine and cyclophosphamide, followed by infusions of haploidentical NK cell and IL-2 [117]. In the 15 patients who underwent Treg depletion using the IL-2-diphtheria fusion protein (IL2DT), the rate of NK cell expansion was 27% compared to 10% in the 42 patients who did not. The use of IL2DT also improved CR rates from 21% to 53%. These results suggested that IL2DT may be able to circumvent host Treg interference of NK cell proliferation and activity.

Similar to IL-2, IL-15 is able to exert stimulatory effects on NK cells but without inducing Treg suppression [116]. Two phase I studies of recombinant human IL-15 in combination with haploidentical NK cell infusions for solid tumors and AML showed improvements in in vivo expansion compared to historical reports of IL-2 mediated expansion [118]. Among 26 patients with AML, 32% achieved a CR. In contrast to IL-2, CRS was observed with the subcutaneous formulation of IL-15 in the solid tumor cohort, but not with the intravenous route in the AML cohort. A phase I first-in-human study of ALT-803, an IL-15 superagonist complex, was carried out in patients with hematologic malignancies who relapsed after allogeneic HSCT, resulting in an ORR of 19% with 1 CR lasting 7 months [119]. This study demonstrated a significant increase in NK and CD8^+^ T cells without a corresponding upregulation of Treg cells.

CAR-NK cells have also been engineered for ectopic IL-15 expression, which has dramatically improved donor NK persistence and proliferation [6,93]. Preclinically, the piggybac transposon system has been used to generate CAR-NKs targeting NKG2D ligands with co-expression of ectopic IL-15, which have in vitro and in vivo activity in AML models [120]. While recent experience with piggybac transposon-edited CAR-T therapies have led to concerns of the development of CAR T-cell lymphomas, insertional mutagenesis has not been demonstrated in follow-up studies [121,122].

Finally, IL-12 is also able to activate NK cells and induce production of IFN-γ [123]. Expansion using IL-12 promotes proliferation of more mature NK cell phenotypes with enhanced ADCC in combination with rituximab [124]. In murine models, pre-activation with IL-12, IL-15, and IL-18 can overcome the resistance of T-cell ALL to autologous NK cell adoptive transfers [125]. Furthermore, as previously discussed, the combination of IL-12, IL-15, and IL-18 can differentiate primary NK cells into memory-like NK cells, improving on the short-term priming effect from IL-2 or IL-15 alone [69,71]. These cytokine-induced memory-like NK cells demonstrated enhanced cytolytic activity and expansion, suggesting a promising platform for CAR- and TCR-based cellular therapies [71,126,127].

### 4.2. NK Cell Checkpoint Blockade

In the TME, both the number and activity of NK cells are suppressed, with many demonstrating a dysregulated and “exhausted” phenotype [128,129,130]. The presence of CD56^dim^ NK cells with an exhausted phenotype have been associated with poorer proliferation and reduced survival in multiple myeloma [131]. Exhausted NK cells display decreased expression of activating receptors such as NKG2D, CD16, and NKp46, along with increased expression of inhibitory receptors including NKG2A [130,132]. Therefore, blockade of inhibitory NK receptors may be useful in circumventing NK exhaustion and enhancing cytotoxicity. Furthermore, tumor cells with preserved MHC expression can still bind to inhibitory NK receptors, providing additional rationale for targeting these NK cell checkpoints.

Preclinical studies of IPH2101, an anti-KIR2D antibody, showed improvement in NK cell-mediated lysis against AML tumor cells while sparing peripheral blood mononuclear cells [133]. This was followed by two phase I studies of IPH2101 as monotherapy and in combination with lenalidomide for the treatment of relapsed or refractory multiple myeloma, showing a good safety profile and some modest response with the combination strategy [134,135]. Unfortunately, IPH2101 failed to demonstrate efficacy in a subsequent phase II investigation for smoldering MM [136]. Lirilumab, another KIR inhibitor that binds the same epitope as IPH2101, was only able to impart mild benefit in AML and MDS when used in combination with azacitidine [137,138]. One hypothesis for the lack of efficacy is the integral role that KIR inhibition plays in the education of NK cells to develop functional competence [139,140]. Monalizumab, an inhibitor of NKG2A, has resulted in improvements in the in vitro cytotoxicity of NK cells against chronic lymphocytic leukemia (CLL) cells [141]. NKG2A is an inhibitory NK receptor that binds to HLA-E, which is over-expressed on CLL B cells [142]. Monalizumab is currently being in the post-allogeneic HSCT setting for hematologic malignancies (NCT02921685).

Non-MHC-specific receptors such as PD-1 have also been found to be upregulated on NK cells in exhausted states, exerting a suppressive effect on their proliferative potential and cytokine secretion [143]. Preclinical evidence suggests that the suppressive effects of the TME may be ameliorated with immune checkpoint inhibition [144,145,146]. A number of trials have assessed PD-1 and CTLA-4 checkpoint inhibition in hematologic malignancies with variable responses but notably minimal efficacy in multiple myeloma and common off-target toxicities [147,148,149,150,151]. Of note, a recent phase II trial evaluating high dose cytarabine with a day 14 infusion of the PD-1 inhibitor pembrolizumab in relapsed or refractory AML demonstrated an encouraging ORR of 46% and a composite complete response rate of 38% [152]. Interestingly, complete response was associated with enrichment of progenitor exhausted CD8^+^ TCF1^+^ T cell populations. A complete treatment of immune checkpoint inhibition is beyond the scope of this article but has been reviewed elsewhere [153].

### 4.3. Modulating the Tumor Microenvironment

Approaches to modulate the suppressive elements of the TME are currently under development. Tumor growth factor β (TGF-β) is a cytokine in the TME that promotes Treg function and are known to play an important role in tumor metastasis [154,155]. TGF-β has been shown to negatively impact NK cells in the microenvironment by inhibiting expression of activating receptors such as NKG2D and reducing IFN-γ production [156,157]. NK cells engineered with TGF-βR2 receptors carrying impaired downstream signaling have demonstrated enhanced cytotoxicity against certain solid tumors, though this has not yet been explored in the hematologic malignancies [158,159]. Clustered regularly interspaced short palindromic repeats (CRISPR)/cas12a gene editing approaches have also been applied to generate iPSC-derived NK cells with disrupted TGF-βR2, allowing for an easily scalable manufacturing platform [160]. Finally, the buildup of adenosine byproducts from upregulated CD73 activity in the hypoxic TME can in turn downregulate NK cell function in the TME [161,162,163]. A phase I study of the anti-CD73 monoclonal antibody, CPI-006, in combination with pembrolizumab or ciforadenant, an adenosine 2A receptor antagonist, is currently ongoing for multiple cancer types including NHL (NCT03454451). Only results from the solid tumor cohorts have been reported thus far, showing modest responses and fair tolerability [164].

### 4.4. Enhancing Antibody-Dependent Cellular Cytotoxicity

Monoclonal antibodies against tumor antigens have shown tremendous success in the treatment of hematologic malignancies. Notably, tumor-bound antibodies can recruit NK cells and induce ADCC, making it a mechanism that may be exploited to enhance NK cell cytotoxicity [165]. The combination of NK cell adoptive transfer with a monoclonal antibody has been evaluated in several settings. A phase I study evaluated nicotinamide-expanded allogeneic NK cells with concurrent rituximab or elotuzumab in NHL and multiple myeloma, respectively [166]. Among the 15 patients with NHL, 10 achieved a CR and 1 had a PR as best response, leading to an ORR of 73%. Similarly, a phase I/II trial of allogeneic NK cells at doses up to 9 × 10^7^ cells/kg resulted in an ORR of 56% with manageable toxicities, though donor-specific antibodies were detected in one patient [167]. Obinutuzumab is an anti-CD20 monoclonal antibody with an afucosylated Fc section that allows greater potency in mediating ADCC when compared to rituximab [168]. Preclinical models have demonstrated profound cytotoxicity against B cell lymphoma cell lines when combining obinutuzumab with cord blood-derived NK cells [169,170]. A phase I study of an iPSC-derived NK cell with high-affinity CD16 is currently ongoing for B cell lymphomas in combination with rituximab or obinutuzumab (NCT04245722).

KIR genotypes may impact the potency of ADCC from NK cells. Patients with follicular lymphoma and expression of KIR2DL2 and KIR3DL1 appear to have improved outcomes with rituximab treatment, suggesting that selection for upregulation of these receptors on NK cells may benefit adoptive transfer strategies [171]. In addition, CRISPR/cas9 gene editing techniques have been applied to induce knock-outs of ADAM17 and knock-ins of high affinity CD16 to enhance NK-mediated ADCC [172,173]. CRISPR-based gene modifying platforms may allow for favorable balancing of activating and inhibiting receptor signaling to maximize effector function in NK cells. Other future directions of interest include the development of an Fc-optimized CD133 antibody and harnessing the immunomodulatory properties of lenalidomide to further augment ADCC of NK cell therapies [174,175].

### 4.5. NK Cell Engagers

Approaches enhancing the engagement of NK cells to target tumor antigens are currently being explored. Similar to commercially available bi-specific antibodies in lymphoid malignancies, these NK cell engagers are able to concurrently bind both tumor antigens and NK cell receptors, thereby facilitating recruitment and cytotoxicity. Bi-specific killer engagers (BiKEs) carry two single chain variable fragments (scFv) that bind to an activating NK receptor such as CD16 and a tumor-specific antigen such as CD19 or CD33, while tri-specific killer engagers (TRiKEs) are BiKEs that contain an additional scFv to further enhance NK cell cytotoxicity [176]. One major advantage of NK cell engagers is that they tend to be less time-consuming and less costly to manufacture compared to CAR-NK cells as they do not require complex gene editing [177].

Preclinical investigations have evaluated and validated the tumor-killing efficacy of a CD16/CD33 BiKE against MDS as well as AFM13, a CD16/CD30 BiKE against CD30+ lymphomas [178,179]. AFM13 binds to an epitope on CD16A that is distinct from the Fc binding site, reducing the risk of steric hindrance with IgG binding [180]. This has recently been translated into a phase Ib study of AFM13 with pembrolizumab in relapsed or refractory Hodgkin lymphoma, which demonstrated an ORR of 83% and a favorable toxicity profile with infusion reactions being the most common adverse event [180]. A subsequent phase I/II clinical trial was carried out to evaluate cord blood-derived memory-like NK cells pre-complexed with AFM13 followed by AFM13 infusions for the treatment of relapsed or refractory CD30+ lymphoma [181]. In all, 19 patients with actively progressing lymphoma received lymphodepletion with fludarabine and cyclophosphamide, followed by NK cell infusions up to 1 × 10^8^ cells/kg and 3 weekly doses of AFM13. This strategy was shown to have a favorable toxicity profile with no evidence of CRS, ICANS, or GVHD. Notably, the ORR was 89% and the CR rate was 42%, despite the lack of any bridging chemotherapy. The presence of donor NK cells was confirmed at up to 1 month, though longer term persistence is still unknown.

TRiKEs have also been developed to simultaneously engage two separate activating NK receptors (e.g., CD16/NKp46/CD19 TRiKEs) or to cross-link proliferative cytokines (e.g., CD16/CD33/IL-15 TRiKEs) [182,183]. The inclusion of an IL-15 linker into an NK cell engager not only enhanced effector function but also improved in vivo persistence and activation through IL-15 [184]. A multicenter phase I/II study of a CD16/CD33/IL-15 TRiKE is ongoing for CD33+ myeloid neoplasms (NCT03214666).

## 5. Conclusions

Recent exciting advances in CAR-T therapies have transformed the therapeutic landscape of hematologic malignancies, but growing experience has made clear the limitations of an autologous cell therapy approach. An increasing interest in allogeneic strategies has prompted a search for alternative immune cell platforms. Early observations on the anti-tumor benefits of KIR mismatch in HSCT has led to investigations of adoptive transfer approaches, which have shown favorable safety profiles in various early phase studies [75,76,86]. Different expansion and activation methods utilizing combinations of supportive cytokines are actively being explored and improved upon. Notably, the induction of a memory-like phenotype using IL-2, IL-15, and IL-18 holds great potential in improving both the cytotoxicity and persistence of NK cells [69]. To date, the most prominent success in NK cell adoptive transfer has been with IL-15-enhanced CAR-NK products against CD19-expressing lymphomas, while progress in other hematologic malignancies and solid tumor targets remain in earlier investigational stages [6,181,185]. Nonetheless, the recent advances in NK cell therapies and other methods in leveraging NK cell cytotoxicity have demonstrated the great promise of this cellular therapy platform.

Several limitations and questions remain. Early clinical trials have reported variable efficacy and differing rates of expansion and persistence. Continuing to improve our understanding of NK cell biology and the factors that lead to an optimal response to NK cell therapy will be critically important. Efforts are underway to enhance NK cell cytotoxicity using a growing armamentarium of gene editing techniques, promote NK persistence by further fine-tuning cytokine expression, and bypass resistance mechanisms through modulation of the suppressive tumor microenvironment. Finally, methods to improve access for patients with rapidly progressive disease is of paramount importance. Utilizing a scalable manufacturing NK cell platform may allow for a true off-the-shelf cell therapy product and result in a dramatic reduction in vein-to-vein time. While currently approved cellular therapy approaches have primarily focused on the adaptive immune system, harnessing the potent anti-tumor capabilities of the innate immune system can open a new avenue of immunotherapeutic advancements and accelerate our progress towards a cure for previously incurable diseases.

## Figures and Tables

**Table 1:** Summary of select ongoing clinical trials evaluating allogeneic NK cellular therapies in hematologic malignancies. NCT: National Clinical Trial number (clinicaltrials.gov), NK: natural killer, AML: acute myeloid leukemia, MDS: myelodysplastic syndrome, MM: multiple myeloma, NHL: non-Hodgkin lymphoma, CLL: chronic lymphocytic leukemia, TLR-9: iPSC: induced pluripotent stem cells, TKI: tyrosine kinase inhibitor, CAR: chimeric antigen receptor, PB: peripheral blood, CB: cord blood, HSCT: hematopoietic stem cell transplantation, PD-1: programmed cell death protein-1, HLA: histocompatibility antigen, KIR: killer Ig-like receptors, DLI: donor lymphocyte infusion, TLR-9: toll-like receptor 9, BCMA: B-cell maturation antigen, NKG2A: NK group 2A, BiKE: bispecific killer engager, TRiKE: trispecific killer engager, IL: interleukin.

Phase	Sponsor	NK Cell Product, Other Antineoplastic Agents	Indications	NCT
NK Cell Therapies with HSCT				
II	M.D. Anderson	CB-derived NK cells personalized based on HLA-KIR genotype between 30–180 days after receiving CB transplant	Hematologic Malignancies	NCT02727803
I/II	Washington University in St. Louis	Cytokine-induced memory-like NK cells in combination with DLI for relapsed disease after allogeneic HSCT	AML	NCT03068819
II	Duke University	NK cell-enriched DLI with or without TLR9 agonist after allogeneic HSCT	Hematologic Malignancies	NCT02452697
I	Celularity Inc.	Placental-derived NK cells after autologous HSCT	MM	NCT04309084
II	M.D. Anderson	CB-derived NK cells with rituximab and autologous HSCT	NHL	NCT03019640
Adoptive Transfer NK Cells (Non-Transplant)				
I/II	Washington University in St. Louis	Memory-like NK cells with IL-2	AML, MDS	NCT01898793
I	Fate Therapeutics	iPSC-NK cells with high-affinity CD16, IL-15 receptor fusion, and CD38 knockout + daratumumab in MM patients only	AML, MM	NCT04614636
I	Duke University	Universal donor-expanded NK cells with membrane-bound IL-21, in combination with TKI	CML	NCT04808115
I/II	Gamida Cell Ltd.	Nicotinamide-expanded PB-derived NK cells + rituximab	NHL	NCT05296525
CAR-NK Therapies				
I/II	Asclepius Technology	NK-92-derived BCMA CAR-NK	MM	NCT03940833
I	Wuhan Union Hospital	CB-derived CD19 CAR-NK with transduced IL-15	ALL, CLL, NHL	NCT04796675
I/II	PersonGen BioTherapeutics	PB-derived CD7 CAR-NK with dual co-stimulatory domains	NHL, leukemia	NCT02742727
I	Century Therapeutics	iPSC-derived CD19 CAR-NK with IL-15, safety switch, and proprietary CRISPR-editing to avoid host rejection	NHL	NCT05336409
I	Hangzhou Cheetah Cell Therapeutics	CB-derived NKG2D CAR-NK	AML	NCT05247957
NK Cell Checkpoint Therapies				
I	Institut Paoli-Calmettes	Anti-NKG2A antibody infused 2 months after allogeneic HLA-matched HSCT with reduced intensity conditioning	Hematologic Malignancies	NCT02921685
I/II	Bristol-Myers Squibb	Anti-PD-1 nivolumab as monotherapy or in combination with ipilimumab, daratumumab, or an anti-KIR2D antibody	Hematologic Malignancies	NCT01592370
II	University of North Carolina	Anti-PD-1 pembrolizumab with high dose cytarabine	AML	NCT02768792
Enhancing Antibody-Dependent Cellular Cytotoxicity				
I	Fate Therapeutics	iPSC-NK cells with high-affinity CD16 in combination with rituximab or obinutuzumab	B-cell lymphoma	NCT04245722
I/II	Biohaven Pharmaceuticals	Autologous memory-like NK cells with BHV-1100 (antibody recruiting molecule) and low-dose IL-2	MM	NCT04634435
NK Cell Engagers				
I/II	M.D. Anderson	CB-derived NK cells precomplexed with CD16/CD30 BiKE AFM13, followed by AFM13 intravenous infusions	CD30+ lymphoma	NCT04074746
I/II	GT Biopharma	CD16/CD33/IL-15 TRiKE monotherapy	MDS, AML	NCT03214666
Modulating the Tumor Microenvironment				
I	Corvus Pharmaceuticals	CPI-006 (anti-CD73 antibody) as monotherapy or in combination with pembrolizumab or ciforadenant	Advanced cancers	NCT03454451

## Data Availability

Not applicable.
